# Progress on the Function and Application of Thymosin β4

**DOI:** 10.3389/fendo.2021.767785

**Published:** 2021-12-21

**Authors:** Yuan Xing, Yumeng Ye, Hongyan Zuo, Yang Li

**Affiliations:** ^1^ Department of Experimental Pathology, Beijing Institute of Radiation Medicine, Beijing, China; ^2^ Department of Pharmacy, The First Affiliated Hospital of Hebei North University, Zhangjiakou, China; ^3^ Academy of Life Sciences, Anhui Medical University, Hefei City, China

**Keywords:** thymosin β4, apoptosis, inflammation, signaling pathway, tissue repair, angiogenesis

## Abstract

Thymosin β4 (Tβ4) is a multifunctional and widely distributed peptide that plays a pivotal role in several physiological and pathological processes in the body, namely, increasing angiogenesis and proliferation and inhibiting apoptosis and inflammation. Moreover, Tβ4 is effectively utilized for several indications in animal experiments or clinical trials, such as myocardial infarction and myocardial ischemia-reperfusion injury, xerophthalmia, liver and renal fibrosis, ulcerative colitis and colon cancer, and skin trauma. Recent studies have reported the potential application of Tβ4 and its underlying mechanisms. The present study reveals the progress regarding functions and applications of Tβ4.

## Introduction

Thymosin is a lymphocyte growth factor that was initially extracted from the calf thymus by Goldstein and White (1). The thymosin family can be divided into three groups: α, β, and γ thymosin, based on the differences in their isoelectric point. The isoelectric point of thymosin β (Tβ) is 5.0–7.0 (2). At present, 15 types of β-thymosin have been identified; of these, three main forms are found in the human body (Tβ4, Tβ10, and Tβ15), Tβ4 being the most abundant, accounting for 70%–80% of β-thymosin (3–5). Tβ4 is found in various tissues, particularly in the thymus, spleen, and peritoneal macrophages (6)and is highly expressed in the brain, liver, kidney, testis, myocardium, platelets, and leukocytes (7).

## Biological Function of Tβ4

Tβ4 comprises 43 amino acids and its biological activity is determined by encoded gene fragments. The first four amino acids of Tβ4 regulate the anti-inflammatory and antifibrotic effects (8, 9), whereas amino acids 1–15 inhibit apoptosis and reduce the toxicity induced damage caused to cells (10). The active fragment encoded by amino acids 17–23 triggers angiogenesis and growth of hair follicles (11, 12).

### Tβ4 Promotes Angiogenesis

Tβ4 promotes angiogenesis, enhances endothelial progenitor cell (EPC) viability, and triggers the proliferation and migration of cells as well as formation of capillary-like structures in cells (13). Vascular endothelial growth factor (VEGF) is an important paracrine factor secreted by the progenitor cells to promote angiogenesis, which can further induce proliferation, differentiation, and migration of endothelial cells and increase vascular permeability. Tβ4 upregulates VEGF expression, i.e., when Tβ4-pretreated EPCs were transplanted into the infarcted rat heart, the expression of VEGF in the border region was markedly increased than that after EPC transplantation alone (14). The combination of Tβ4 and human adipose-derived stem cells was used to treat hindlimb ischemia in mice. Moreover, Tβ4 enhances the endothelial differentiation of these stem cells by upregulating various angiogenic factors, such as angiopoietin-1 and von Willebrand factor; furthermore, it triggers blood perfusion and collateral formation in the hindlimb by increasing the capillary density (15).

### Effects of Tβ4 on Cell Proliferation and the Cell Cycle

Tβ4 affects the cell cycle and promotes cell proliferation. After knocking-out Tβ4 in intestinal epithelial cells, cells slowly proliferated, cell cycle was affected indicating a marked decrease in the G0/G1 population and a remarkable increase in polyploid populations among these cells, and DNA replication was affected by DNA damage (16). Moreover, intrahippocampal infusion of N-acetyl-erythritosyl-lysyl proline (a Tβ4 peptide) facilitates the generation of new neurons in the hippocampus (17). Tβ4 treatment enhances the proliferation of mesenchymal stem cells (MSCs), particularly those derived from adjacent adipose tissue, and interleukin (IL-8) crucially mediates Tβ4-enhanced proliferation (18). Furthermore, Tβ4 enhances the proliferation of oligodendrocyte progenitor cells (OPCs) and their maturation into myelinating oligodendrocytes (19). Furthermore, it stimulates the proliferation of adult rat cardiac progenitor cells and promotes their differentiation into vascular endothelial cells, coronary smooth muscle cells, and cardiomyocytes (20). Additionally, Tβ4 accelerates vascular endothelial cell proliferation, thereby protecting post-ischemic cardiac function (21).

### Tβ4 Inhibits Apoptosis

Tβ4 treatment alleviated tubular epithelial cell apoptosis by inhibiting the transforming growth factor (TGF)-β pathway in Sprague-Dawley (SD) rats with chronic renal tubular interstitial fibrosis (22). Moreover, it prevents nucleus pulposus cell apoptosis, reduces cellular aging, and promotes cell proliferation (23). Tβ4 further decreased the apoptosis rate of EPCs induced by serum depletion and markedly downregulated the expression of the apoptosis-related proteins caspase-3 and caspase-9 in EPCs (24). Furthermore, Tβ4 prevented mitochondrial disruption and inhibited caspase-mediated apoptosis of human corneal epithelial cells exposed to ethanol *in vitro*, indicating that it may function as an antiapoptotic agent (25). In addition, Tβ4 may inhibit neuronal apoptosis by upregulating glucose-regulated protein 78 and downregulating C/EBP homologous protein and caspase−12, thereby reducing cerebral ischemia/reperfusion injury (26). In oxygen-glucose deprived and reoxygenated (OGD/R) cells, the rate of apoptosis was increased and GRP78, CHOP, and Bax were upregulated; however, Bcl-2 was downregulated, which was reversed by Tβ4 overexpression. Moreover, Tβ4 prevented OGD/R-induced endoplasmic reticulum stress-dependent apoptosis in cortical neurons (27). As Tβ4 could attenuate the OGD/R-associated downregulation of P62 and Bcl-2 as well as the upregulation of autophagy mediators, such as autophagy-related protein-5 and the ratio of microtubule-associated protein 1 light chain 3, it effectively inhibited PC12 cell apoptosis and autophagy induced by OGD/R (28). Moreover, Tβ4 treatment upregulated the expression of miR-200a; however, the increase in miR-200a downregulated the expression of p53 and reduced apoptosis of progenitor cells subjected to oxygen glucose deprivation (OGD) (29).

### Tβ4 Ameliorates Inflammation

Tβ4 ameliorates inflammatory reactions. In a mouse model of autoimmune encephalomyelitis, hematoxylin-eosin staining showed markedly decreased the number of inflammatory cells in the brains of Tβ4-treated mice (30). In models of liver injury mediated by ethanol and lipopolysaccharide, Tβ4 prevented the activation of nuclear factor kappa B (NF-κB) by blocking the phosphorylation of the inhibitory protein IκB, thereby preventing the production of proinflammatory cytokines such as tumor necrosis factor-α (TNF-α), IL-1β, and IL-6 (31). A neonatal mouse fetal alcohol spectrum disorder model revealed that Tβ4 treatment effectively blocked the increase in ethanol-induced inflammatory factors and decreased the expression of TNF-α and IL-1β (32).

## Tβ4 and Signaling Pathways

**Figure 1:**
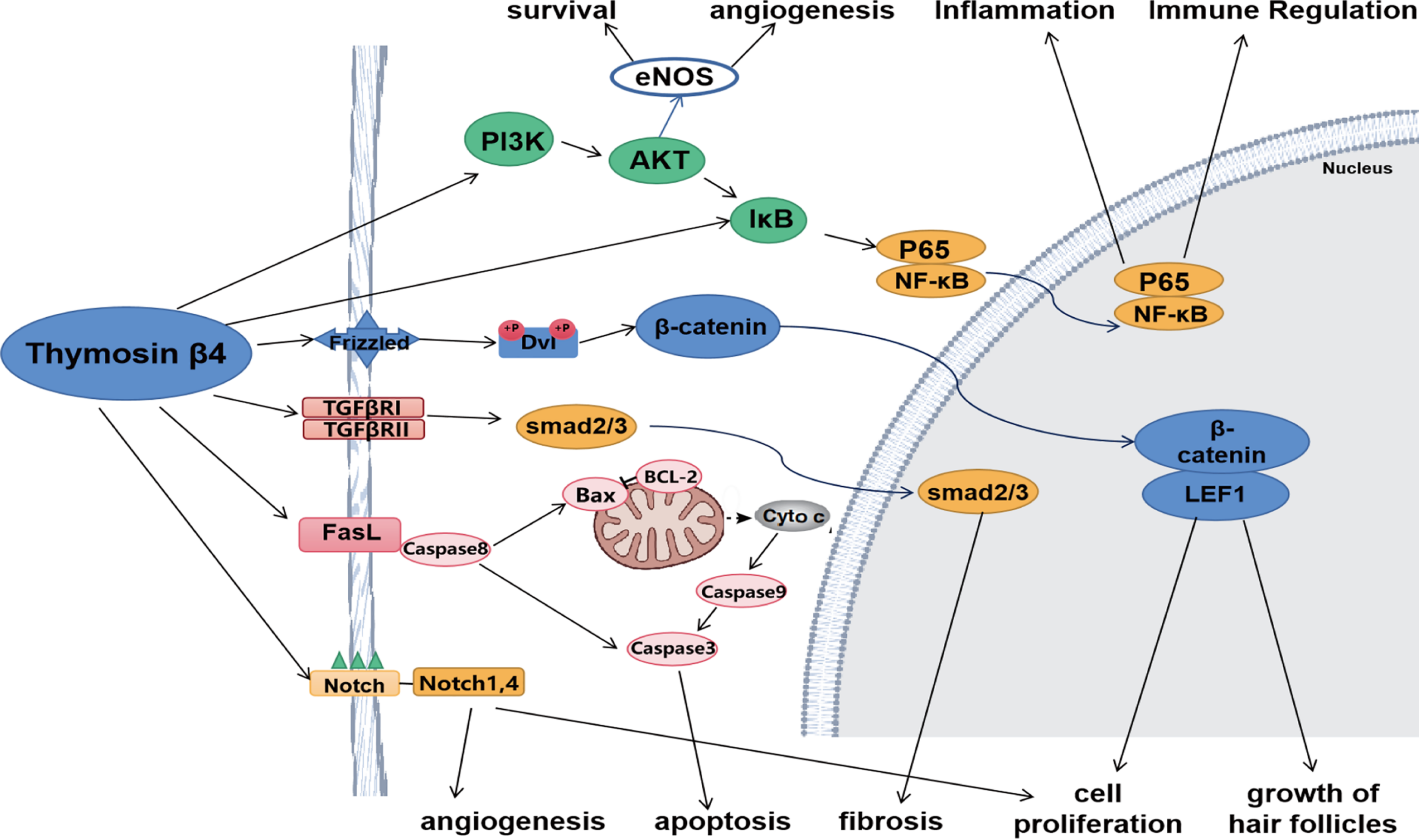
Tβ4 regulates various signaling pathways. Tβ4 ameliorates inflammatory damage by regulating NF-κB and Toll-like receptor pathways. During tissue repair, Tβ4 regulates PI3K/Akt/eNOS and Notch pathways. In addition, Tβ4 regulates TGF-β pathway to alleviate fibrosis and Wnt pathway to promote hair follicle formation. Tβ4 also regulates apoptosis pathway to inhibit apoptosis.

Tβ4 affects the secretion of multiple cytokines and regulates various signaling pathways. It alleviates inflammatory damage by regulating the NF-κB and Toll-like receptor pathways and reducing the release of cytokines such as TNF-α and IL-1 receptor-associated kinases. During tissue repair, Tβ4 regulates PI3K/Akt/eNOS, Notch, angiopoietin-1/Tie2, and other pathways. In addition, it also regulates various signaling pathways, such as the TGF-β pathway to attenuate fibrosis and the Wnt pathway to promote hair follicle generation (**Figure 1**).

### PI3K/Akt/eNOS Pathway

PI3K/Akt is an important pathway associated with microangiogenesis, and plays a pivotal role in cell migration, cell survival, and angiogenesis (33, 34). PI3K/Akt is the upstream pathway of eNOS and affects its transcription and translation. eNOS increases the local mobilization of EPCs and participates in angiogenesis (35). Moreover, exogenous Tβ4 stimulates EPC proliferation, migration, and adhesion *via* the PI3K/Akt/eNOS signal transduction pathway (36). After intraperitoneal injection of Tβ4 in rats with cerebral ischemia and reperfusion, the level of Akt phosphorylation and the expression of eNOS in the cerebral cortex increased, regeneration of blood vessels around the infarction occurred, and the neurological function of the rats was recovered (37). Furthermore, Tβ4 induces angiogenesis *via* PI3K/AKT signaling pathway in ischemic limb diseases (38). Systemic injection of a Tβ4-specific C-terminal tetrapeptide enhanced the early myocyte survival by activating Akt-mediated signaling, increased coronary vessel growth, and inhibited inflammation in mice and pigs (39).

### Notch Pathway

The Notch signaling pathway comprises four Notch receptors (Notch-1, 2, 3, 4, and 5) and five ligands (40). This pathway is crucial in neuronal function, tumor cell proliferation, apoptosis, angiogenesis, arterial endothelial cell stability, and expansion of bone marrow hematopoietic stem cells (41). Tβ4 induces angiogenesis in human umbilical vein endothelial cells (HUVECs) *via* Notch signaling pathway. In the presence of Tβ4, the expression of Notch1 and Notch4 increased in a dose- and time-dependent manner and the speed of lumen formation was accelerated. When the Notch pathway is inhibited, the efficacy of Tβ4 decreases (42). Moreover, Tβ4 inhibited the proliferation and activation of hepatic stellate cells (HSCs), attenuated liver fibrosis by inhibiting Notch signaling, and markedly reduced expression levels of Notch2 and Notch3, which were increased in the liver cells (42). Furthermore, Tβ4 enhanced HUVEC viability, angiogenesis, and migration, as well as promoted the expression of angiopoietin 2, VEGF A, Notch3, and other cytokines in HUVECs in a mouse model of critical limb ischemia (43). In addition, Takeshitak et al. (44) reported that endothelial-specific Notch1 knockdown mice had impaired neovascularization after hindlimb ischemia, and Notch1 induced angiogenesis without VEGF involvement (45). Recent studies reported that Notch signaling could also work in conjunction with VEGF and regulate VEGF expression (46–48). Shu Min et al. reported that Notch1 and Notch4 were required for Tb4-induced VEGF expression and angiogenesis. The downregulation of Notch1 or Notch4 by siRNA or DAPT inhibited Tb4-induced VEGF expression (42).

### TGFβ/Smad Pathway

The TGFβ/Smad signaling pathway is crucially mediated by TGFβ. In a model of fibrosis, TGFβ1 plays an important role in HSC activation (49). TGFβ1 initiates intracellular signal transduction by binding to the TGFβ receptor type II (TGFβR II), and activates TGFβ receptor type I (TGFβR I) kinase. Thereafter, TGFβR I kinase activates the downstream proteins Smad2 and Smad3 *via* phosphorylation. Subsequently, Smad2, Smad3, and Smad4 form a complex and are transferred to the nucleus, where they increase the expression of various fibrotic genes, such as type I and type II collagen, tissue inhibitors of metalloproteinase-1 and -2 (TIMP-1 and TIMP-2), and plasminogen activator inhibitor (PAI)-1 (50). Chen et al. reported that Tβ4 reduced the expression of TGF- β1, TGFβR II, Smad2, and Smad3 in the liver tissues of mice with bile duct ligation. Moreover, they demonstrated that Tβ4 reduced TGFβR II expression level in human hepatic stellate cells LX-2 *in vitro*. These results indicated that Tβ4 alleviated cholestatic liver fibrosis by inhibiting the TGFβ/Smad pathway (51). Zhang et al. reported that Tβ4 treatment markedly inhibited the TGFβ1/KF-κB signaling pathway, which affects neuroprotection and neurorestoration after traumatic brain injury (52).

### Wnt Signaling Pathway

The Wnt signaling pathway is crucially associated with cell proliferation and differentiation and is functionally important for hair follicle morphogenesis (53). In the Wnt signaling pathway, Wnt ligands induce the phosphorylation of Disheveled to prevent GSK3β-dependent phosphorylation of β-catenin (54). β-catenin and lymphokine-1 (Lef-1) are two key molecules in the Wnt signaling pathway (55). Gao et al. reported that Tβ4 stimulated Wnt ligands on the cytomembrane to transmit the signal to accumulate unphosphorylated β-catenin for phosphorylation of Disheveled into the cytoplasm, which further leads to the accumulation of unphosphorylated β-catenin (56). In an epidermal-specific Tβ4-overexpressing mouse model and Tβ4 global knockout mice, changes in β-catenin and Lef-1 expression were similar to those of Tβ4 (56). β-catenin plays a pivotal role in hair follicle growth. After Tβ4 treatment, the number of hair follicles in the mice significantly increased. Moreover, Tβ4 can accelerate hair growth *via* Wnt signaling pathway by elevating the mRNA levels of β-catenin and Lef-1 (57, 58). Additionally, Tβ4 activates the Wnt/catenin signaling pathway in limb progenitor cells and promotes limb regeneration in a frog model (59). Furthermore, it protected Ang II-induced cardiomyocyte growth by regulating the Wnt pathway and Ang II stimulation, thereby leading to myocardial hypertrophy in mice. After Tβ4 treatment, the cardiomyocyte area decreased, and the expression of hypertrophic marker genes, such as atrial natriuretic peptide, b-myosin heavy chain, β-catenin, and Wnt-mediated secretory protein-1, was decreased (60).

### Apoptosis Pathway

The biological mechanism of apoptosis is extremely complex, involving the interaction of numerous proteins with signal transducers and signaling pathways. Members of the Bcl-2 protein family are responsible for regulating apoptosis (61). Previous studies have demonstrated that Tβ4 decreases apoptosis by increasing antiapoptotic proteins and reducing the Bax/BCL2 ratio (62). Sosne et al. demonstrated that Tβ4 treatment decreased deleterious mitochondrial alterations, significantly decreased cytochrome c release from mitochondria, and increased Bcl-2 expression in ethanol-exposed human corneal epithelial cells, wherein it inhibited the caspase-2, -3, -8, and -9 activity, with caspase-8 exhibiting highest inhibition (63). Furthermore, FasL-mediated activation of caspases-8 and -3, as well as H(_2_)O(_2_)-triggered stimulation of caspases-9 and -3 in human corneal epithelial T (HCE-T) cells was abolished by preincubating them with Tβ4 (64). Furthermore, Iguchi et al. combined the antitumor drugs with other drugs that interact with apoptotic processes, and found that after apoptosis, a low molecular weight protein, identified to be Tβ4 by HPLC analysis, was commonly decreased, and the morphology of actin filaments changed into clump formations. These results indicate that decreased Tβ4 expression induces apoptosis by antitumor drugs (65).

## Applications of Tβ4 and the Underlying Mechanisms

Due to its rich biological activity and anti-inflammatory effects, Tβ4 regulates several inflammatory cytokines and chemokines and exerts therapeutic effects on various injuries or diseases such as corneal injury, xerophthalmia, and ulcerative colitis. Tβ4 can reduce tissue fibrosis and can be used to treat pulmonary hypertension, pulmonary fibrosis, liver fibrosis, and renal fibrosis. Moreover, it can improve liver function and reduce glomerular injury. Tβ4 can promote angiogenesis, tissue repair, and regeneration, and reduce scar formation. Furthermore, it can be used to promote wound healing, treat myocardial infarction and hindlimb ischemia, and heal damaged ligaments. In addition, Tβ4 exhibits a strong antioxidant effect and can be used to treat cerebral or myocardial ischemia-reperfusion injury.

### Protective Effect of Tβ4 on the Heart

Myocardial infarction (MI) leads to sudden heart attack, and occurs during inappropriate flow of blood to a part of the heart, thereby causing injury to the heart due to lack of oxygen supply (66). MI has a high rate of disability and mortality, and is the leading cause of cardiac death (67).

Tβ4 reduces the infarct size and improves contractile performance in chronic myocardial ischemic injury through two phases: an acute phase that occurs immediately after injury, in which Tβ4 preserves the ischemic myocardium *via* antiapoptotic or anti-inflammatory mechanisms, and a chronic phase, in which Tβ4 activates the growth of vascular or cardiac progenitor cells (68). The clinical phase I trial evaluated the safety, tolerability, and pharmacokinetics of single and multiple intravenous injections of Tβ4 in healthy volunteers. No dose-limiting toxicities or serious adverse events were observed. The tendency of terminal clearance in each dose group was consistent, and there was no obvious accumulation after continuous administration (69). These results were in accordance to those of another phase I clinical trial conducted by Ruff et al, wherein they evaluated the safety, incidence of treatment-emergent adverse events, and pharmacokinetic parameters of synthetic Tβ4. Similarly, no dose-limiting toxicities or serious adverse events were observed (70). Subsequently, a phase II clinical trial was conducted in patients with acute myocardial infarction, which confirmed that Tβ4 could protect and repair the heart and reduce the volume of scars after heart attack (71). In addition, Stromberg et al. conducted a safety trial of Tβ4 in children less than one year of age; thereafter, they conducted a randomized, double-blind clinical trial of Tβ4 and placebo during congenital heart surgery. They evaluated the postoperative time to resolution of organ failure, development of low cardiac output syndrome, and echocardiographic index of cardiac dysfunction. These results confirm the clinical utility of Tβ4 in improving ischemia-reperfusion injury during congenital heart surgery (72).

Additionally, Tβ4 attenuates rejection of the transplanted heart after heart transplantation. Tβ4 in combination with adenovirus-associated vector 2.9 was used to treat rejection after heart transplantation. In this case, Tβ4 reduced acute rejection, elevated the density of cardiac capillaries, increase survival rates of miniature pigs after heart transplantation, and markedly enhanced the local myocardial function of the grafts (73).

In addition, Tβ4 exhibits auxiliary functions, such as enhancing the therapeutic effect of MSCs and increasing the time of cardiac regeneration. Under hypoxic conditions, Tβ4 (1 µg/mL) reduces the injury, apoptosis, and caspase-8 activity of MSCs; however, it increases B-cell lymphoma-XL protein expression and MSC proliferation. In an *in vivo* experiment, the injection of MSCs containing Tβ4 into the rat myocardium effectively restored the cardiac function after myocardial infarction, increased cardiac blood flow, and significantly improved survival rates of MSCs (74).

Tβ4 prolongs the time of heart regeneration in mammals. Hearts of 1-day-old mice regenerated after partial surgical resection, and this effect was lost by 7 days of age; however, Tβ4 could extend the cardiac regeneration potential of neonatal mice to the 7th postnatal day (75).

Transplantation of EPCs can repair the heart *via* angiogenesis or secretion of protective paracrine factors (76); however, transplantation of autologous EPCs has numerous limitations, including the limited supply of expanded EPCs, impaired function, and activity of the transplanted cells (77). Transplantation of Tβ4-pretreated EPCs to the injured heart could treat acute ST-segment elevation in myocardial infarction. The cardiac function of the Tβ4 group was significantly improved compared with that of the control group, and no serious complications were observed (78).

Considering the underlying mechanisms of protective role of Tβ4 in heart injuries, previous studies reported that treatment with Tβ4 in the myocardial infarction setting improves cardiac function by activating Akt phosphorylation, promoting the ILK-Pinch-Parvin complex, and suppressing NF-κB. Furthermore, Tβ4 selectively upregulates catalase, Cu/Zn-SOD, and Bcl2, thereby protecting cardiac fibroblasts from H_2_O_2_ induced oxidative damage (79). In the myocardial infarction model, Tβ4 enhanced cardiac function by suppressing NF-κB, thereby attenuating cardiac fibrosis (80, 81).

In summary, Tβ4 exerts therapeutic effects on various heart-related diseases such as myocardial infarction and myocardial ischemia-reperfusion injury, indicating that it may be used as a promising drug for the clinical treatment of heart diseases in the future.

### Therapeutic Effects of Tβ4 on Corneal Injury and Dry Eye Syndrome

Recent studies have reported that Tβ4 exerts a therapeutic effect on corneal injury and dry eye syndrome. Corneal injuries are common in chemical burns and oxidative injuries. Some chemicals can quickly lead to corneal stromal dissolution, activate stromal fibroblasts, cause a large amount of inflammatory cell infiltration, and eventually lead to corneal ulcers and perforations, which can lead to blindness (82).

Tβ4 can affect the secretion of numerous cytokines, promote corneal re-epithelialization, dampen untoward inflammation, and inhibit apoptosis; thus, it exerts therapeutic effect on corneal injury (83). Moreover, it can reduce corneal inflammation and regulate the balance of cellular matrix metalloproteinases and tissue inhibitors of metalloproteinases, thus promoting corneal wound repair after alkaline injury and improving corneal transparency. In various corneal injury models, such as chemical injury and corneal epithelial debridement, Tβ4 exhibited strong anti-inflammatory and wound healing effects (84). Recombinant Tβ4 treatment of corneal burns in rabbits revealed that recombinant Tβ4 effectively promoted newborn tissue remodeling and corneal burn repair, as recombinant Tβ4 regulates the expression of MMP-2 and TIMP-2 to promote tissue repair (85). In a model of hydrogen peroxide-induced oxidative corneal injury, Tβ4 promoted the growth and migration of rabbit corneal epithelial cells, reduced apoptosis, enhanced antioxidant capacity, and exerted a strong protective effect on damaged corneas (86).

Dry eye syndrome is a common ophthalmic disease characterized by ocular surface inflammation (87). Tβ4 can slow eye dryness and accelerate wound healing. It markedly alleviated xerophthalmia symptoms in a mouse model. A randomized double-blind clinical phase II trial revealed that the Tβ4 treatment group revealed a 35.1% reduction in ocular discomfort than that of the vehicle control group and a 59.1% reduction in total corneal fluorescein staining than that of the vehicle control group. Other improvements observed in Tβ4–treated patients included tear film breakup time and increased tear volume production (88). Furthermore, glycine Tβ4 eye drops significantly increased conjunctival goblet T cells, significantly decreased corneal cell apoptosis, and reduced inflammatory cytokine levels and T cells in the conjunctiva (89). A randomized double-blind clinical phase II trial conducted by Sosne et al. revealed that eye discomfort in the Tβ4 treatment group was reduced by 35.1% than that in the control group, and the total corneal fluorescein staining was reduced by 59.1%. In addition, improvement in tear film breakup time and increase in tear volume was observed in Tβ4–treated patients (88).

### Tβ4 Promotes Skin Wound Healing

Wound healing includes angiogenesis, cell proliferation, differentiation, migration, epithelial reconstruction, and wound closure, by various cytokines. Although the design of skin flaps and surgical techniques are constantly improving, ischemic necrosis remains a common clinical problem (90). Tβ4 can promote cell migration and angiogenesis, regulate various cytokines, such as intercellular adhesion molecule (ICAM-1), MMP, laminin (LN), VEGF, and basic fibroblast growth factor, inhibit apoptosis, eliminate inflammation, and reduce oxidative damage (91, 92). Moreover, it can increase cell migration in various injury models, particularly the migration of keratinocytes, which cover the wound and protect from fluid loss and infection (81, 93–96). Male Sprague-Dawley rats were subjected to random-pattern skin flap operations. Tβ4 significantly reduced necrotic areas; rats that received 5 mg/kg Tβ4 twice per day presented the highest survival rates. VEGF expression and superoxide dismutase activity were markedly increased, whereas malondialdehyde levels were reduced (97). In a full-thickness skin defect Sprague-Dawley rats rat model, VEGF and basic fibroblast growth factor revealed sustained and stable high expression after treatment with recombinant Tβ4, which inhibited LN-5 expression in the early stage, beneficial for cell proliferation and differentiation; furthermore, it upregulated LN-5 expression in the middle and late stages, which was beneficial for improving the matrix environment and promoting epidermal cell migration and wound healing (98). Additionally, Tβ4 improved burn wound healing and promoted angiogenesis and wound closure, which may be associated with the long-term expression of heat shock protein 70, related to F-actin regulation during the wound-healing period (99). In addition, Tβ4 associates actin polymerization with metalloproteinase synthesis to promote cell migration. One mechanism proposes that profilin-dependent dissociation of the G-actin-Tβ4 complex liberates actin for filament assembly (96). Tβ4 binds to integrin-linked kinase in the lamellipodia to activate Akt2 and increase metalloproteinase production (100). Moreover, it increases laminin-332 synthesis, which is a known migration factor for various epithelial and endothelial cells, including keratinocytes (101, 102). Collectively, Tβ4 has the potential to heal and regenerate dermal injuries, and been successfully used in several clinical trials. Fine et al. organized a randomized double-blind clinical trial to determine whether Tβ4 may be beneficial in promoting wound healing in patients with epidermolysis bullosa (EB). A solitary noninfected cutaneous wound of standardized size was treated on a daily basis with either one of three doses of Tβ4 or a placebo control. Simultaneously, the occurrence of adverse effects was sought to confirm the safety of Tβ4 when applied to EB skin, both in children and adults. Furthermore, the occurrence of adverse reactions was studied to confirm the safety of Tβ4 when applied to EB skin. Although it has not been proven, topical Tβ4 may be an extremely important supplement in the overall management of patients with this potentially devastating disease (103). Phase II clinical trials for the use of Tβ4 in epidermolysis bullosa, pressure sores, and venous stasis ulcers have been completed. Treadwel et al. organized 143 patients with chronic cutaneous (stage III/IV) pressure ulcers (full thickness) and venous stasis ulcers; results revealed that Tβ4 accelerated healing by almost a month in patients who healed (104). Another double-blind, placebo-controlled, dose-escalation study was conducted at eight locations in Europe. This study recruited 73 randomly assigned patients. The study reported that Tβ4 had the potential to accelerate wound healing, and approximately 25% of patients could heal completely within 3 months, particularly those with small to moderate wounds (105).

### Protective Effect of Tβ4 on the Liver

Tβ4 does not bind to heparin; therefore, it can spread freely into the tissue. It ameliorated carbon tetrachloride (CCl4)-induced acute liver injury in mice in a dose- and time-dependent manner by suppressing oxidative stress, inhibiting the inflammatory response, and reducing hepatocellular apoptosis (106). Moreover, Tβ4 prevented ethanol- and lipopolysaccharide-mediated oxidative stress by decreasing reactive oxygen species and lipid peroxidation, increasing antioxidant levels, and reducing glutathione and manganese-dependent superoxide dismutase.

Liver fibrosis typically occurs in response to hepatic injury. It is characterized by collagen and extracellular matrix protein deposition in the liver tissues (107). Activated HSCs are responsible for collagen deposition and play a pivotal role in hepatic fibrogenesis (108). Several studies have reported that Tβ4 treatment has an antifibrotic effect on the liver (51, 109–111). Li et al. found that Tβ4 could markedly reduce hydroxyproline content and collagen deposition in the livers of CCl4-induced mice and rats, and relieve liver and pseudo-lobule necrosis, whereas the inhibition of NF-κB p65 might be an underlying mechanism (112). Chen et al. reported that in bile duct ligation mice, exogenous Tβ4 treatment reduced collagen deposition and suppressed α-SMA expression, a marker of HSC activation, indicating that exogenous Tβ4 treatment hindered HSC activation to inhibit cholestatic liver fibrosis (51). Barnaeva et al. (109) demonstrated that Tβ4-treated HSCs upregulated HGF and downregulated PDGF-β receptor at the RNA level. Reyes-Gordillo et al. reported that Tβ4 treatment prevented PDGF-ββ-dependent proliferation and migration of cultured human HSCs by inhibiting PDGF-ββ-dependent phosphorylation of AKT. They found that Tβ4 interrupted the movement of AKT into PI3K, blocking the phosphorylation of AKT by PI3K in HSCs treated with PDGF-ββ (110).

### Tβ4 Promotes Hair Growth

In recent years, Tβ4 has been closely related to hair follicle development and hair growth. Topical application of Tβ4 promotes hair growth in rats and mice and it stimulates early differentiation of rat epithelial progenitor cells (113). Moreover, Tβ4 may act on hair follicle reconstruction by upregulating fibronectin expression in human dermal papilla cells (114). After shaving, the hair of Tβ4-overexpressing transgenic mice grew faster and longer than those of wild-type mice (115). Moreover, in aged mice with sparse hair, Tβ4 accelerated hair growth for more than 26 weeks. After topical administration of Tβ4, the hair of nude mice grew faster and thicker than those of normal mice (113). Tβ4 accelerates hair growth by increasing the proliferation of outer hair follicle root sheath cells, that is, hair follicles grew better and proliferated faster in the Tβ4 group than in the control group when the outer hair follicle root sheath cells were cultured *in vitro* (116).

### Tβ4 Alleviates Renal Fibrosis

Chronic kidney disease is characterized by abnormalities in renal structure or function that last for more than 3 months and has an adverse impact on the health of the patient (117). Endogenous Tβ4 is dispensable in healthy kidneys. In contrast, a lack of endogenous Tβ4 exacerbated symptoms in mouse models of glomerular disease and angiotensin II-induced renal injury. The administration of exogenous Tβ4 or its metabolite Ac-SDKP revealed therapeutic effects various experimental models of kidney disease, such as glomerulonephritis, diabetic nephropathy, and hypertensive nephropathy (118). In renal fibrosis, Tβ4 is upregulated in glomerulosclerosis and is required for the angiotensin II-induced expression of plasminogen activator inhibitor-1) PAI-1 (119). In addition, Tβ4 treatment might alleviate renal fibrosis and tubular epithelial cell apoptosis by inhibiting the TGF-β pathway in rats with unilateral ureteral obstruction and chronic renal tubular interstitial fibrosis (22).

### Effect of Tβ4 on Ulcerative Colitis and Colon Cancer

Tβ4 is expressed in the human intestine, where it modulates the intestinal immune system (120). Moreover, it is considered to be effective in treating gastrointestinal disorders (121). In a mouse colitis model resembling Crohn’s disease, AAV-Tβ4-treated mice displayed distinctly attenuated colon injuries and reduced the apoptosis rates in colonic mucosal epithelia. AAV-Tβ4 significantly reduced inflammatory cell infiltration and alleviated oxidative stress in the inflamed colons of mice, as evidenced by decreased myeloperoxidase activity and malondialdehyde levels and increased superoxide dismutase activity. AAV-Tβ4 further modulated colonic TNF-α, IL-1β, and IL-10 levels and suppressed the compensatory proliferation of colonic epithelial cells (122).

In addition, Tβ4 exerts a therapeutic effect on colon cancer. The expression of Tβ4 in rectal cancer stem cells was higher than that in normal epithelial cells. Lentivirus was used to reduce levels of Tβ4 in rectal cancer stem cells, and interestingly, this treatment reduced the tumor size and aggressiveness of colorectal cancer stem cell-based xenografts in mice (123).

### Tβ4 Alleviates Inflammation

Tβ4 exhibits anti-inflammatory activities in different pathologies (124) and reduces inflammation in the brain (26), liver (27), eye (89), and heart diseases (80). Diverse mechanisms underlying the inflammatory response *via* Tβ4 regulation following injuries are observed (125). NF-κB regulates the expression of various inflammatory genes and is crucial in the inflammatory process (126). Tβ4 can downregulate NF-κB (127) and reduce levels of numerous inflammatory cytokines such as TNF-α (128). It can also prevent the activation of NF-κB by blocking the phosphorylation of the inhibitory protein IκB, thereby inhibiting proinflammatory cytokine production (31). Sosne et al. demonstrated that in human epithelial corneal cells stimulated with TNF-α, Tβ4 significantly decreased NF-κB activation, p65 subunit phosphorylation, and nuclear translocation (127). Ping et al. reported that Tβ4 could inhibit TNF-α-induced NF-κB activation and block RelA/p65 translocation and the sensitizing effects of its intracellular binding partners PINCH-1 and integrin-linked kinase (128). Furthermore, some studies have provided preliminary evidence on the ability of Tβ4 to resolve inflammation by promoting noncanonical autophagy associated with the activation of the DAP kinase anti-inflammatory function (129–132).

In summary, Tβ4 exerts therapeutic effects on various injuries or diseases of different tissues, while the underlying mechanisms have some similarities and differences (**Tables 1** and **2**).

**Table 1:** Actions of Tβ4 and mechanisms.

Encoded gene fragments	Actions	Target tissue	Indications	Mechanism	References
1-4 amino acids	anti-inflammatory	brain	autoimmune encephalomyelitis	suppresses the secretion of interleukin-8 and the activation of NF-κB significantly.	(30)
1-4 amino acids	anti-inflammatory	liver	ethanol- and LPS-induced liver injury	,inhibits the activation of NF-κB pathway, thereby preventing the production of proinflammatory cytokines, such as tumor necrosis TNF-α, IL-1β, and IL-6.	(31)
1-4 amino acids	anti-inflammatory	brain	fetal alcohol spectrum disorders	attenuates p38, ERK MAPKs, and NF-B pathway activation, and enhance miR-339-5p expression induced by ethanol exposure in microglia.	(32)
1-4 amino acids	anti-inflammatory	eye	dry eye syndrome	reduces IL-1β, IL-6, TNF-αand IFN-γ and CD4^+^/CCR5^+^T cells.	(89)
1-4 amino acids	anti-inflammatory	liver	hepatic ischemia-reperfusion injury	activates AKT-Bad pathway and inhibits the expression of TNF-α and IL-6.	(97)
1-4 amino acids	anti-fibrosis	liver	liver fibrosis	inhibits the Notch signaling, reduces the expression of NF-κB p65, inhibits PDGF-β-dependent phosphorylation of AKT, and interrupts the movement of AKT into PI3K.	(110, 112, 133)
1-4 amino acids	anti- fibrosis	kidney	renal fibrosis	inhibits the TGF-β pathway.	(22)
1-15 amino acids	anti-apoptosis	brain	cerebral ischemia/reperfusion injury	upregulates GRP78 and downregulates CHOP and caspase-12.	(26)
1-15 amino acids	anti-apoptosis	brain	diseases associated with demyelination disorders	up-regulates miR-200a, increases MBP synthesis after targeting Grb2 and thereby inactivating c-Jun from inhibition of MBP synthesis; and inhibits OGD-mediated apoptosis after targeting EGFR inhibitor (Mig-6), PI3K inhibitors (FOG2 and Pten) and an inducer (p53) of pro-apoptotic genes, for AKT activation and down-regulation of p53.	(29)
1-15 amino acids	anti-apoptosis	heart	myocardial infarction	reduces caspase-8 activity, increases Bcl-XL protein expression.	(74)
1-15 amino acids	anti-apoptosis	heart	cardiovascular disorders	decreases the expression and activity of caspase-3 and -9, which markedly increased the Bcl-2/Bax ratio, and ILK-Akt activation.	(24)
1-15 amino acids	anti-apoptosis	eye	corneal diseases	decreases FasL-mediated activation of caspases-8 and -3 as well as H(2)O(2)-triggered stimulation of caspases-9 and -3.	(64)
1-15 amino acids	anti-apoptosis	eye	vision disorder	inhibits caspase-2, -3, -8, and -9 activity.	(63)
1-15 amino acids	anti-apoptosis	colon	Crohn’s disease	decreases TNF-α, IL-1β and IL-10 and decreases MPO activity and MDA content, increases SOD activity.	(122)
17 – 23 amino acids	promotes hair growth	hair follicle	depilation	accelerates hair growth through the Wnt signaling pathway by increasing the mRNA levels of β-catenin and Lef-1.	(58)
17 – 23 amino acids	improves wound healing	skin	full-thickness skin defect SD rat model	regulates VEGF, bFGF and LN-5.	(98)
17 – 23 amino acids	improves wound healing	skin	mouse burn model	Upregulates the expression of heat-shock proteins (HSP70), p-AKT and VEGF signaling pathways.	(99)
17 – 23 amino acids	stimulates angiogenesis	heart	myocardial infarction	upregulates the expression of VEGF, activates Akt-mediated signaling, promotes the ILK-Pinch-Parvin complex, and suppresses NF-κB.	(14, 39, 79)
17 – 23 amino acids	stimulates angiogenesis	hind limb	hindlimb ischemia	upregulates various angiogenic factors, such as angiopoietin-1 and von Willebrand factor, activates the PI3K/AKT signaling pathway, promotes the expression of angiopoietin2, VEGFA, Notch3 and other cytokines in HUVECs	(15, 38, 43)
17 – 23 amino acids	stimulates angiogenesis	brain	cerebral ischemia and reperfusion	increases the level of Akt phosphorylation and the expression of eNOS in the cerebral cortex, and regenerates blood vessels around the infarction.	(37)
40 – 43 amino acids	increases heart function post-ischemia	heart	rat model of acute myocardial ischemia-reperfusion	decreases the level of MDA in serum and myocardial tissue and increases the activity of SOD.	(132)

**Table 2:** Clinical trials of Tβ4.

Phase	Drug	Indications	Participants	Dosage regimen	Conclusions	Status	References
I	chemosynthetic Thymosin β4	acute myocardial infarction	40 healthy volunteers	42, 140, 420, or 1260 mg/kg intravenous injections for 14 days.	There were no dose limiting toxicities or serious adverse events.	completed	(70)
I	Recombinant Human Thymosin β4	acute myocardial infarction	54 healthy volunteers	0.05, 0.25, 0.5, 2.0, 5.0, 12.5 or 25.0 μg/kg in the single-dose intravenous injections trial and in the multiple-dose intravenous injections trial, 0.5, 2.0 and 5.0 μg/kg were administered once rh-Tβ4 daily for 10 days	It was well tolerated and safe in healthy people and suitable for use in a clinical study for the treatment of acute myocardial infarction.	completed	(69)
II	chemosynthetic Thymosin β4	acute myocardial infarction	patients with acute myocardial infarction	Not report	Tβ4 could protect and repair the heart and reduce the volume of scars after heart attack.	completed	(71)
II	chemosynthetic Thymosin β4	congenital heart surgery	12 children up to four months of age	Tβ4 at 5 mg/kg/dose, 12.5 mg/kg/dose, and 20 mg/kg/dose, intravenous injections, and given in the operating room 15–30 minutes before cardiopulmonary bypass	Tβ4 could improve ischemia-reperfusion injury during congenital heart surgery.	completed	(72)
II	RGN-259 (Thymosin β4)	dry eye	9 patients with severe dry eye	Each 8 mL plastic squeeze bottle contained 2.0 mL fill volume	Tβ4 could tear increase film breakup time and tear volume production.	completed	(88)
II	chemosynthetic Thymosin β4	epidermolysis bullosa	Approximately 35–40 patients with RDEB or JEB, aged 2 or above	Not report	Although as yet unproven, topically applied Tβ4 may prove to be an extremely important addition to the overall management of patients with this potentially devastating disease.	Recruiting	(103)
II	Thymosin β4 Gel	stasis and pressure ulcers	143 total patients with chronic cutaneous (stage III/IV) pressure ulcers (full thickness) and venous stasis ulcers	0.01%, 0.03%, or 0.1% Tβ4 in the gel formulation	Tβ4 could accelerate healing by almost a month in those patients that did heal.	completed	(104)
II	Thymosin β4 Gel	venous stasis ulcers	73 patients	0.01%, 0.03%, or 0.1% Tβ4 in the gel formulation and treated for 84 days	Tβ4 could accelerate wound healing and that complete wound healing can be achieved within 3 months in about 25% of the patients.	completed	(105)

## Conclusion and Future Perspectives

Tβ4 is a natural endogenous repair factor that is activated during development and tissue damage. This peptide exerts various biological activities, such as inhibition of inflammation and apoptosis as well as promotion of proliferation and angiogenesis. Moreover, animal experiments and clinical studies have reported that Tβ4 exerts therapeutic effects on several diseases or injuries, such as myocardial infarction and myocardial ischemia-reperfusion injury, xerophthalmia, liver and renal fibrosis, ulcerative colitis and colon cancer, and skin trauma. The regulation of Tβ4 in some signaling pathways, including the PI3K/Akt/eNOS pathway, Notch pathway, TGFβ/smad pathway, Wnt pathway, and apoptosis pathway, might serve as the underlying mechanisms of its effects.

Judging from the updated literature outlined in this review, there is burgeoning interest in the functions and applications of Tβ4. It could be speculated that Tβ4 might be a safe and efficacious new drug for various clinical indications in the near future. However, the remarkable progress in both basic research and clinical trials has also raised new questions, such as thorough elucidation of the mechanisms of some applications, and exploration of more promising indications. The upstream and downstream components regulate Tβ4 functions need to be thoroughly delineated, especially the upstream regulation mechanism. Moreover, the crosstalk of the downstream signaling pathways of Tβ4 should be further clarified. Furthermore, some functions of Tβ4, such as the inhibition of inflammation and apoptosis as well as promotion of proliferation and angiogenesis, should be explored furtherly to find new clinical indications. Currently, our group and others are conducting preclinical studies to demonstrate the efficacy of Tβ4 in various animal models, with the goal of pushing new indications into clinical trials. In addition, new technologies in pharmacy, pharmaceutics and material science should be used to promote the application of Tβ4 in different indications in a more appropriate dosage form.

## Author Contributions

All authors contributed to draft and revise the article, gave final approval of the version to be published, and agreed to be accountable for all aspects of the work.

## Funding

This study was supported by the funds from Major national science and technology projects (Grant No. 2018ZX09J18103-006).

## Conflict of Interest

The authors declare that the research was conducted in the absence of any commercial or financial relationships that could be construed as a potential conflict of interest.

## Publisher’s Note

All claims expressed in this article are solely those of the authors and do not necessarily represent those of their affiliated organizations, or those of the publisher, the editors and the reviewers. Any product that may be evaluated in this article, or claim that may be made by its manufacturer, is not guaranteed or endorsed by the publisher.
